# Genetic diversity and transmission pattern of multidrug-resistant tuberculosis based on whole-genome sequencing in Wuhan, China

**DOI:** 10.3389/fpubh.2025.1442987

**Published:** 2025-01-28

**Authors:** Liqing Wei, Jun Chen, Zhen Deng, Zefang Zhang, Zhengbin Zhang, Qionghong Duan

**Affiliations:** ^1^Department of Blood Transfusion, Wuhan Wuchang Hospital, Wuhan University of Science and Technology, Wuhan, China; ^2^Department of Clinical Laboratory, Wuhan Pulmonary Hospital, Wuhan, China; ^3^Department of Tuberculosis Prevention and Control, Wuhan Pulmonary Hospital, Wuhan, Hubei, China

**Keywords:** multidrug-resistant tuberculosis, whole-genome sequencing, genetic diversity, transmission pattern, Wuhan, China

## Abstract

**Background:**

Investigating the molecular epidemiological characteristics of multidrug-resistant tuberculosis (MDR-TB) in China’s moderate-burden regions, such as Wuhan, is crucial for understanding and controlling disease transmission.

**Materials and methods:**

This study analyzed MDR-TB isolates from pulmonary tuberculosis cases registered at Wuhan Pulmonary Hospital in 2017. Whole genome sequencing (WGS) was used to identify resistance-conferring mutations, examine their associations with specific *Mycobacterium tuberculosis* lineages or sublineages, and assess clustering profiles.

**Results:**

Among the 149 analyzed strains, the most prevalent mutations associated with resistance to 11 anti-tuberculosis drugs were identified as follows: *rpoB* Ser450Leu (59.73%, rifampicin), katG Ser315Thr (62.42%, isoniazid), embB Met306Val (42.86%, ethambutol), rpsL Lys43Arg (68.13%, streptomycin), pncA Trp68Arg (10.53%, pyrazinamide), gyrA Asp94Gly (22.50%, fluoroquinolones), and rrs 1401A > G (50.00–100.00%, second-line injectable aminoglycosides). Additional mutations were detected in fabG1 c-15C > T (42.86%, ethionamide) and thyX c-16C > T (21.43%, p-aminosalicylic acid). Notably, rare mutations absent from the WHO mutation catalog, such as ahpC c-52C > T and rpsL Lys43Thr, were also observed. The mutation frequency of *embB* Met306Ile was significantly higher in Lineage 4 (L4) strains than in Lineage 2 (L2) strains (*p* = 0.0150), while the rpsL Lys43Arg mutation frequency was lower in L4 compared to L2 (*p* = 0.0333). A total of 31 MDR MTB *Mycobacterium tuberculosis* isolates formed clusters, resulting in a clustering rate of 20.81% and a recent transmission rate of 11.41%. The clustering rates between L4 and L2 strains were not significantly different (χ^2^ = 0.0017, *p* > 0.05).

**Conclusion:**

The genetic diversity of MDR-TB in Wuhan demonstrates unique characteristics, with evidence of localized transmission. These findings highlight the urgent need to strengthen measures to detect early cases of MDR-TB and control transmission of MDR-TB in the region.

## Introduction

According to the World Health Organization (WHO) Global Tuberculosis Report 2024, an estimated 0.40 million individuals worldwide were affected by multidrug- or rifampicin-resistant tuberculosis (MDR/RR-TB) in 2023, with only 44% of cases enrolled in treatment programs (1). For the 2021 patient cohort (the most recent with available data), the global treatment success rate for MDR/RR-TB was 68%. These figures underscore the persistent challenge that MDR/RR-TB poses to global public health (1).

China reported an estimated 741,000 new tuberculosis (TB) cases in 2023, with an incidence rate of 52 per 100,000 population. While the country’s TB incidence ranks at the lower-middle level globally, it remains the third-largest contributor to the global TB burden, accounting for approximately 6.8% of total cases. The WHO classifies China as a high-burden country for TB, AIDS-related TB, and MDR/RR-TB (1).

Wuhan, a major transportation hub and the fourth-largest city in China with a resident population exceeding 10 million, is also the economic center of central China. It hosts the second-largest number of colleges and universities nationwide, contributing to a complex population structure that impacts TB transmission. In 2023, the TB notification rate in Wuhan closely mirrored the national average (43.65 vs. 43.49 per 100,000 population), as reported by the National Tuberculosis Program system (2).

Rapid and accurate detection of drug-resistant TB is essential for controlling the epidemic. While phenotypic drug susceptibility testing (DST) remains the gold standard, its diagnostic timeframe of 10 days to 2 months limits its effectiveness in rapid intervention. Molecular diagnostic tools, such as GeneXpert MTB/RIF, have significantly reduced diagnostic time but are restricted to detecting mutations at high-frequency loci with variable sensitivity and specificity across regions (3, 4). Whole-genome sequencing (WGS) offers a comprehensive solution by detecting all known mutations in drug resistance genes with high sensitivity and specificity, enabling significant reductions in diagnostic time (5, 6).

Beyond diagnostics, WGS has proven to be an effective tool for genotyping, cluster identification, and studying the transmission dynamics of TB (7, 8). While alternative genotyping methods such as insertion sequence 6,110-restriction fragment length polymorphism (IS6110-RFLP) (9), spoligotyping (10), and mycobacterial interspersed repetitive unit-variable number tandem repeat (MIRU-VNTR) analysis can provide insights (11), they are limited in scope and lack the accuracy offered by WGS. Consequently, the accuracy of these genotyping methods is limited compared to the comprehensive resolution offered by WGS (12).

Drug resistance mutations and clustering of *Mycobacterium tuberculosis* isolates are influenced by phylogenetic lineages, which can also be elucidated through WGS. To date, no studies have explored the genetic diversity and recent transmission dynamics of multidrug-resistant tuberculosis in Wuhan using WGS. Our previous research (13) utilized a combination of 24-locus MIRU-VNTR, deletion-targeted multiplex polymerase chain reaction (DTM-PCR), and sequencing of resistance-associated genes to genotype MDR-TB isolates from Wuhan Pulmonary Hospital in 2017. This study utilized WGS data to analyze the spectrum of drug resistance mutations, identify clusters, and examine transmission patterns in Wuhan.

## Materials and methods

### Isolates from registered cases

In 2017, 341 MDR-TB patients were registered in the Wuhan TB reporting network, a component of the National Tuberculosis Program system. Of these, 151 cases were identified at Wuhan Pulmonary Hospital, one of two designated tuberculosis treatment centers in Wuhan. After excluding two isolates that were unavailable, a total of 149 isolates were included in this study and preserved in the strain library at −80°C using glycerol-containing 7H9 medium. These 149 subcultures were subjected to WGS after their initial isolation.

### DST

DST was performed using the proportion method on the Lowenstein-Jensen medium (Baso Company, China). Susceptibility to a total of eleven anti-tuberculosis (anti-TB) drugs, comprising five first-line and six second-line drugs, was assessed in accordance with WHO guidelines (14). The H37Rv strain was used as a quality control reference throughout the testing process.

### WGS

**Figure 1:**
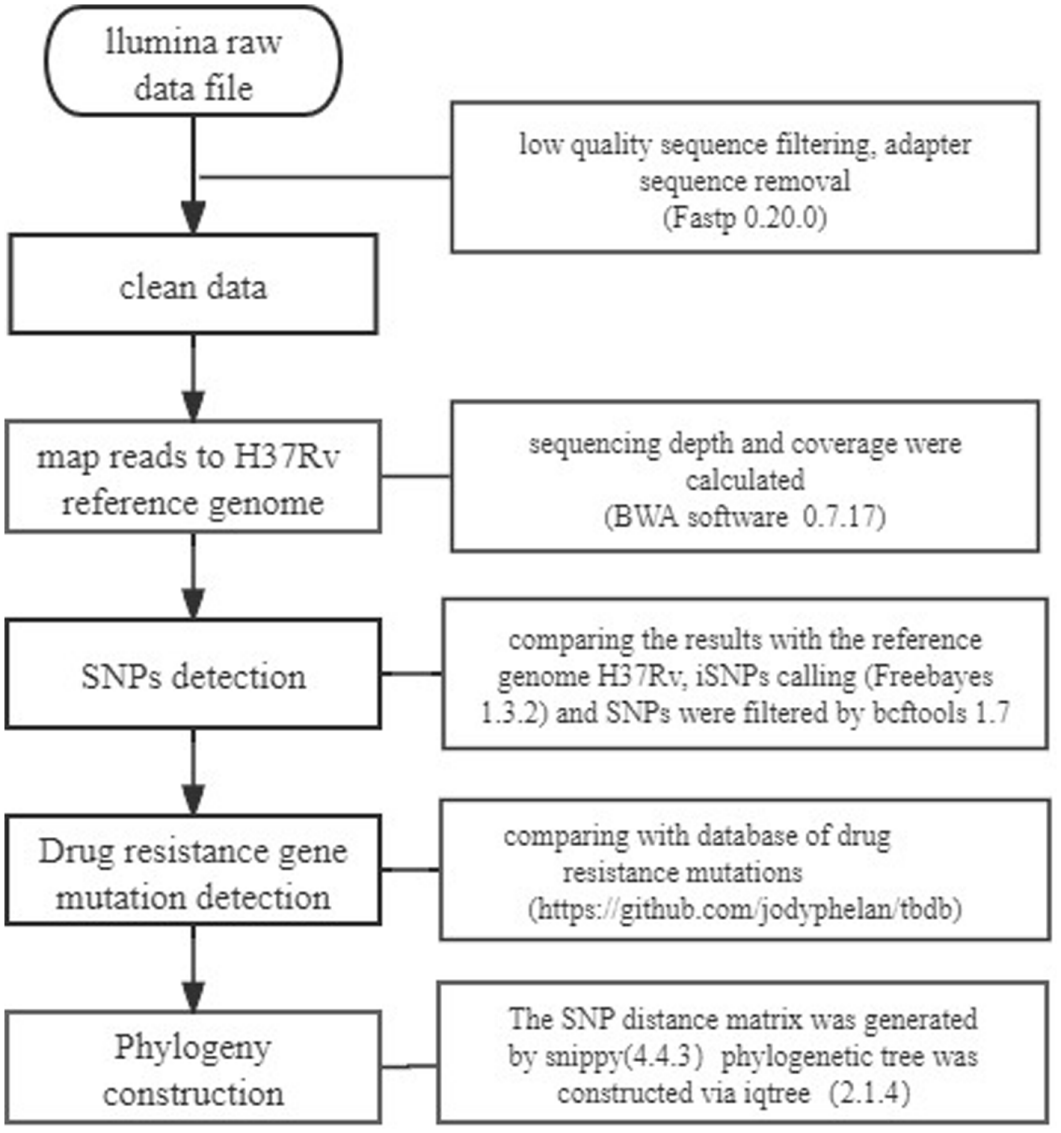
Flowchart depicting the bioinformatics data analysis pipeline for identification of SNPs and drug resistance gene mutations from raw sequencing reads.

WGS of 149 MDR-TB clinical isolates was outsourced to Shanghai Jingnuo Biotechnology Co., Ltd., which provided the raw sequencing data. Sequencing was performed using the Illumina NovaSeq 6,000 platform (Illumina, United States) with paired-end sequencing (PE150). The bioinformatics pipeline used for analyzing raw sequencing reads, including the identification of single nucleotide polymorphisms (SNPs) and drug resistance gene mutations, is outlined in Figure 1.

### WGS data collation and cleaning

The WGS data collation and cleaning process involved the following steps: Raw sequencing reads from 149 strains were processed using Fastp (version 0.20.0) to automatically remove reads containing adaptor sequences or those of low quality. High-quality reads were then aligned to the reference genome of the standard *Mycobacterium tuberculosis* strain H37Rv (GenBank accession: NC_000962.3), obtained from the NIH GenBank database. Alignment was performed using BWA software (version 0.7.17), and sequencing depth and genome coverage were calculated. Next, sequencing data were filtered based on specific thresholds: sequencing depth > 100× and genome coverage >95%. From the processed data, 149 datasets met these criteria, with an average sequencing depth of 205.9× and genome coverage of 99.29%. These datasets were deemed suitable for further analysis.

### Identification of single nucleotide polymorphisms

SNPs were identified by aligning the sequencing data against the reference genome *Mycobacterium tuberculosis* H37Rv. SNP calling was conducted using Freebayes (version 1.3.2).

To ensure data quality, candidate SNPs were filtered using Bcftools (version 1.7). Filters excluded SNPs with sequencing depth below 10 or mapping quality scores less than 100. As a result, a refined set of high-confidence SNPs was obtained for downstream analysis.

### Drug resistance gene mutation detection

Whole-genome SNPs identified in this study were compared against a database of known drug-resistance mutations.^1^ This comparison facilitated the characterization of drug-resistance gene mutations in each strain. Additionally, these mutations were analyzed to predict the likelihood of drug resistance in the isolates.

### Phylogeny construction

The mutation matrix for each strain was generated using Snippy software (v4.4.3) based on identified mutation sites. From this mutation matrix, an SNP distance matrix was computed to determine the genetic relationships between strains. Strains with an SNP difference ≤ 12 were identified as clustered. A maximum-likelihood phylogenetic tree was then constructed using core SNPs via IQ-TREE software (v2.1.4).

### Cluster definition

In this study, genomic clusters were defined based on an SNP cutoff value of ≤12, as established in the majority of prior studies (15, 16). The minimum estimate of the recent infection rate was calculated using the formula (13) $\left(n_{c}-c\right)/n$ , where $n_{c}$ represents the total number of clustered isolates, c is the number of clusters, and n represents the total number of isolates analyzed.

### Modern Beijing sublineage definition

The Modern Beijing sublineage was identified by the presence of both the C1477596T (ogt12) mutation and a single copy of IS6110 in the NTF region (17).

### Statistical analysis

Statistical analyses were conducted using SPSS version 21.0 (SPSS, United States). The Pearson chi-square test or Fisher’s exact probability test was applied to assess the statistical significance of differences in rates (e.g., mutation frequency) between groups, with a significance level of *α* = 0.05. Gene mutation frequencies below 3% were excluded from the analysis tables to ensure robust data representation.

### Ethics statement

Ethical approval for this study was granted by the Wuhan Pulmonary Hospital Medical Ethics Committee (protocol number: 2018-12). Written informed consent was obtained from all tuberculosis patients prior to their enrollment in the study.

## Results

### Demographic and clinical features

Of the 149 TB patients with MDR *Mycobacterium tuberculosis* isolates, 109 (73.15%) were male, and 40 (26.85%) were female. The age of patients ranged from 14 to 88 years, with a mean age of 46.53 years.

The associations between demographic characteristics (sex and age), clinical features (treatment history and sputum smear results), and the lineage of MDR-MTB isolates were analyzed and are summarized in Table 1.

**Table 1:** Demographic and clinical features of patients infected with L4 and L2 lineage isolates.

	Number of L4 (*n* = 7)	Number of L2 (*n* = 142)	χ^2^	*p*
Sex				
Male	6	103		
Female	1	39	0.1098	0.7404
Age (years)				
14–60	2	111		
≥60	5	31	6.4537	0.0111
Treatment history				
New case	4	91		
Retreated	3	51	0.0009	0.9763
Positive sputum smear result				
No	1	37		
Yes	6	105	0.0642	0.8000

### Drug-resistance mutation spectrum

Table 2 summarizes the drug-resistance mutation spectrum of 149 MDR-MTB isolates, highlighting mutations in resistant strains, while Supplementary Table S1 provides comprehensive details on the drug-resistance mutations, lineage information, and DST results.

**Table 2:** Drug resistance gene mutation (for resistant isolates) spectrum of 149 MDR-MTB strains.

Drug	Number of resistant strains (%)	Mutation type	Number of mutant strains	Mutation Percentage (%)	WHO-classification
Rifampicin (RIF)	149 (100.00)	rpoB_p.Ser450Leu	89	59.73	Assoc w R
		rpoB_p.Asp435Val	8	5.37	Assoc w R
		rpoB_p.His445Asp	7	4.70	Assoc w R
		rpoB_p.Leu452Pro	6	4.03	Assoc w R
		rpoB_p.His445Arg	4	2.68	Assoc w R
		rpoB_p.His445Tyr	4	2.68	Assoc w R
		rpoB_p.Leu430Pro, rpoB_p.His445Gln	3	2.01	Assoc w R, Assoc w R- Interim
		rpoC_p.Leu527Val, rpoB_p.Ser450Leu	3	2.01	NA, Assoc w R
		rpoB_p.Ser450Trp	3	2.01	Assoc w R
		rpoB_p.Asn437Asp, rpoB_p.Asp435Val	2	1.34	Assoc w R- Interim, Assoc w R
		rpoB_p.Asp435Tyr	2	1.34	Assoc w R
		rpoB_p.His445Leu	2	1.34	Assoc w R
Isoniazid (INH)	149 (100.00)	katG_p.Ser315Thr	93	62.42	Assoc w R
		fabG1_c.-15C>T	12	8.05	Assoc w R
		ahpC_c.-52C>T	4	2.68	NA
		katG_p.Ser315Asn	4	2.68	Assoc w R
		katG_p.Ser315Thr, fabG1_c.-15C>T	3	2.01	Assoc w R, Assoc w R
		inhA_p.Ile194Thr, fabG1_c.-15C>T	2	1.34	NA, Assoc w R
		katG_c.-7G>A	2	1.34	NA
		katG_p.Ser315Ile	2	1.34	Assoc w R- Interim
		ahpC_c.-81C>T	2	1.34	NA
Ethambutol (EMB)	49 (32.89)	embB_p.Met306Val	21	42.86	Assoc w R
		embB_p.Met306Ile	5	10.20	Assoc w R
		embB_p.Asp354Ala	2	4.08	Assoc w R
Streptomycin (SM)	91 (61.07)	rpsL_p.Lys43Arg	62	68.13	Assoc w R
		rpsL_p.Lys88Arg	9	9.89	Assoc w R
		rrs_r.514A>C	4	4.40	Assoc w R
		rpsL_p.Lys43Thr, rrs_r.514A>C	2	2.20	NA
		rrs_r.517C>T	2	2.20	Assoc w R
Pyrazinamide (PZA)	19 (12.75)	pncA_p.Trp68Arg	2	10.53	Assoc w R
		pncA_p.Gln10Pro	1	5.26	Assoc w R
		pncA_p.His51Tyr	1	5.26	Assoc w R
		pncA_p.Ser67Pro	1	5.26	Assoc w R
		pncA_c.-11A>G	1	5.26	Assoc w R
		pncA_p.Tyr103*	1	5.26	Assoc w R
		pncA_c.171_172insA	1	5.26	NA
		pncA_p.His71Arg	1	5.26	Assoc w R*
Ofloxacin (OFX)	40 (26.85)	gyrA_p.Asp94Gly	9	22.50	Assoc w R*
		gyrA_p.Ala90Val	8	20.00	Assoc w R*
		gyrA_p.Ser91Pro	5	12.50	Assoc w R*
		gyrA_p.Asp94Ala	2	5.00	Assoc w R*
		gyrA_p.Asp94Asn	2	5.00	Assoc w R*
		gyrA_p.Asp94Gly, gyrA_p.Ala90Val	2	5.00	Assoc w R*, Assoc w R*
Amikacin (AMK)	4 (2.69)	rrs_r.1401A>G	4	100.00	Assoc w R
Kanamycin (KAN)	5 (3.36)	rrs_r.1401A>G	4	80.00	Assoc w R
Capreomycin (CAP)	6 (4.03)	rrs_r.1401A>G	3	50.00	Assoc w R
Ethionamide (ETO)	7 (4.70)	fabG1_c.-15C>T	3	42.86	Assoc w R
		inhA_p.Ile194Thr, ethA_c.197_198insT	2	28.57	NA, NA
		inhA_p.Ile194Thr, fabG1_c.-15C>T	1	14.29	NA, NA
P-aminosalicylic acid (PAS)	14 (9.40)	thyX_c.-16C>T	3	21.43	PAS is mentioned in the WHO mutational catalogue
		folC_p.Ile43Thr	2	14.29	
		folC_p.Ser150Gly	1	7.14	
		folC_p.Glu40Gly	1	7.14	
		folC_p.Glu153Ala, thyA_p.His75Asn	1	7.14	
		thyA_p.His75Asn, folC_p.Ser150Gly	1	7.14	
		thyX_c.-16C>T, thyA_p.Trp83Cys	1	7.14	

Frequency of mutant less than 3% are not listed in the table. Assoc w R = Associated with Resistance (group 1); Assoc w R- Interim = Associated with Resistance interim (group 2); NA = Not Available. *for Levofloxacin.

### Rifampicin resistance

Among 145 RIF-resistant isolates (97.32%, 145/149), 97.93% (142/145) harbored mutations in the rifampicin-resistance-determining region (RRDR) of the rpoB gene. The most prevalent mutation was rpoB Ser450Leu (59.73%), followed by Asp435Val, His445Asp., Leu452Pro, and others (Supplementary Table S1). Combined mutations at two sites were observed in 15 strains (10.07%, 15/149), while 5 strains (3.35%, 5/149) exhibited compensatory mutations in the rpoC gene (Supplementary Table S1).

### Isoniazid (INH) resistance

Mutations associated with INH resistance were detected in 138 strains (92.62%, 138/149), with 23 mutation types identified. The dominant mutation was katG Ser315Thr (62.42%, 93/149), followed by fabG1 c-15C > T, ahpC c-52C > T, and katG Ser315Asn. Mutations were mainly associated with katG (77.18%, 115/149) and fabG1 (15.44%, 23/149). Combined mutations were observed in 12 strains (8.05%, 12/149) (Supplementary Table S1).

### Ethambutol resistance

Among 49 EMB-resistant strains (26.53%, 49/149), 13 mutation types were identified. Common mutations included embB Met306Val (42.86%, 21/49) and Met306Ile (10.20%, 5/49). Combined mutations at two sites were observed in 3 strains (6.12%, 3/49) (Supplementary Table S1).

### Streptomycin resistance

SM resistance was identified in 80 strains (87.91%, 80/91), with six mutation types primarily involving the rpsL and rrs genes. The most frequent mutations were rpsL Lys43Arg (68.13%, 62/91) and Lys88Arg (9.89%, 9/91). Combined mutations were observed in 2 strains (2.20%, 2/91) (Supplementary Table S1).

### Pyrazinamide resistance resistance

PZA resistance mutations were located in the pncA gene and its promoter region. Eight mutation types were identified, with pncA Trp68Arg (10.53%, 2/19) being the most frequent, followed by Gln10Pro, His51Tyr, Ser67Pro, and others.

### Second-line drug resistance

Second-Line Drug Resistance: Ofloxacin (OFX): Among 40 OFX-resistant strains, 10 mutation types were identified, with a high mutation rate in the quinolone-resistance-determining region (QRDR) of 96.88% (31/32).

Dominant mutations included gyrA Asp94Gly (22.50%, 9/40), Ala90Val (20.00%, 8/40), and Ser91Pro (12.50%, 5/40). Combined mutations were found in 4 strains (10.00%, 4/40).

Second-Line Injectable Drugs (AMK, KAN, CAP): Mutations primarily occurred in the *rrs* gene, with the 1401A>G mutation observed in all resistant strains: AMK (100.00%, 4/4), KAN (80.00%, 4/5), and CAP (50.00%, 3/6).

### Ethionamide resistance

Ethionamide (ETO): Mutations were identified in the fabG1, ethA, and inhA genes, with the most frequent being fabG1 -15C > T (42.86%, 3/7). Combined mutations were found in 3 strains (42.86%, 3/7).

### Para-Aminosalicylic acid resistance

Para-Aminosalicylic Acid (PAS): Mutations were detected in the thyA, folC, and thyX genes. The dominant mutation was thyX c-16C > T (21.43%, 3/14), with combined mutations observed in 3 strains (21.43%, 3/14).

### Identification of the lineage and sublineage

This study identified two primary lineages among the 149 MDR-TB isolates. Lineage 4 (Euro-American genotype) accounted for 4.70% (7/149) of the isolates, while Lineage 2 (East Asian genotype) was predominant, comprising 95.30% (142/149). Among the isolates belonging to Lineage 2, the Beijing genotype (Lineage 2.2) was the dominant sublineage, representing 95.30% (142/149) of all isolates. In contrast, sublineages Lineage 4.2 and Lineage 4.4 were less common, accounting for 2.68% (4/149) and 2.01% (3/149), respectively. The Beijing genotype strains were further classified into two types: modern Beijing type, which constituted 88.03% (125/142) of the Beijing genotype isolates, and ancient Beijing type, which accounted for 11.97% (17/142). These findings highlight the predominance of the East Asian Lineage 2, particularly the modern Beijing genotype, among the MDR-TB isolates in this study.

### Correlation between mutation in drug resistance-related genes and lineage/sublineage

The embB Met306Ile mutation was significantly more prevalent in lineage 4 compared to lineage 2 (*p* = 0.0150). In contrast, the rpsL Lys43Arg mutation occurred less frequently in lineage 4 (*p* = 0.0333) (Table 3).

**Table 3:** Distribution of main drug resistance gene mutation type in lineages and sublineages of 149 MDR-TB isolates in Wuhan.

Drug	Mutation type	L2(142)		χ^2^	p_1_	L4(7)	χ^2^	p_2_
		Ancient Beijing type (17)	Modern Beijing type (125)					
RIF	rpoB Ser450Leu	7 (41.18)	78 (62.40)	2.8054	0.0939	4 (57.14)	0.0633	0.8013
INH	katG Ser315Thr	13 (76.47)	75 (60.00)	1.7226	0.1894	5 (71.43)	0.0109	0.9167
	fabG1 c-15C>T	1 (5.88)	11 (8.80)	0.0035	0.9530	0 (0.00)		1.000*
EMB	embB Met306Val	6 (35.29)	23 (18.40)	1.6914	0.1934	0 (0.00)	0.7113	0.3990
	embB Met306Ile	1 (5.88)	9 (7.20)	0.0936	0.7596	3 (42.86)		**0.0150** ^*^
SM	rpsL Lys43Arg	8 (47.06)	61 (48.80)	0.0182	0.8928	0 (0.00)	4.5315	**0.0333**
	rpsL Lys88Arg	3 (17.65)	6 (4.80)	2.2779	0.1312	0 (0.00)		1.0000*
PZA	pncA_c-11A>G	0 (0.00)	5 (4.00)		1.0000*	0 (0.00)		1.0000*
OFX	gyrA Asp94Gly	3 (17.65)	5 (4.00)		0.055*	1 (14.29)		0.3595*
	gyrA Ala90Val	0 (0.00)	12 (9.60)	0.7577	0.3840	0 (0.00)		1.0000*
SLID**	rrs 1401A>G	2 (11.76)	5 (4.00)		0.1977*	0 (0.00)		1.0000*
ETO	fabG1 c-15C>T	1 (5.88)	19 (15.20)	0.4417	0.5063	0 (0.00)		0.2378*
PAS	folC Ile43Thr	1 (5.88)	4 (3.20)		0.4766*	0 (0.00)		1.0000*
	thyX-16C>T	0 (0.00)	3 (2.40)		1.0000*	0 (0.00)		1.0000*

*fisher’s exact probability test. **second-line injectable drugs. p_1_:Comparison of ancient Beijing and modern Beijing, p_2_:Comparison of L2 and L4.Bold *p* value means *p* < 0.05.

### Identification of the clustering features

**Figure 2:**
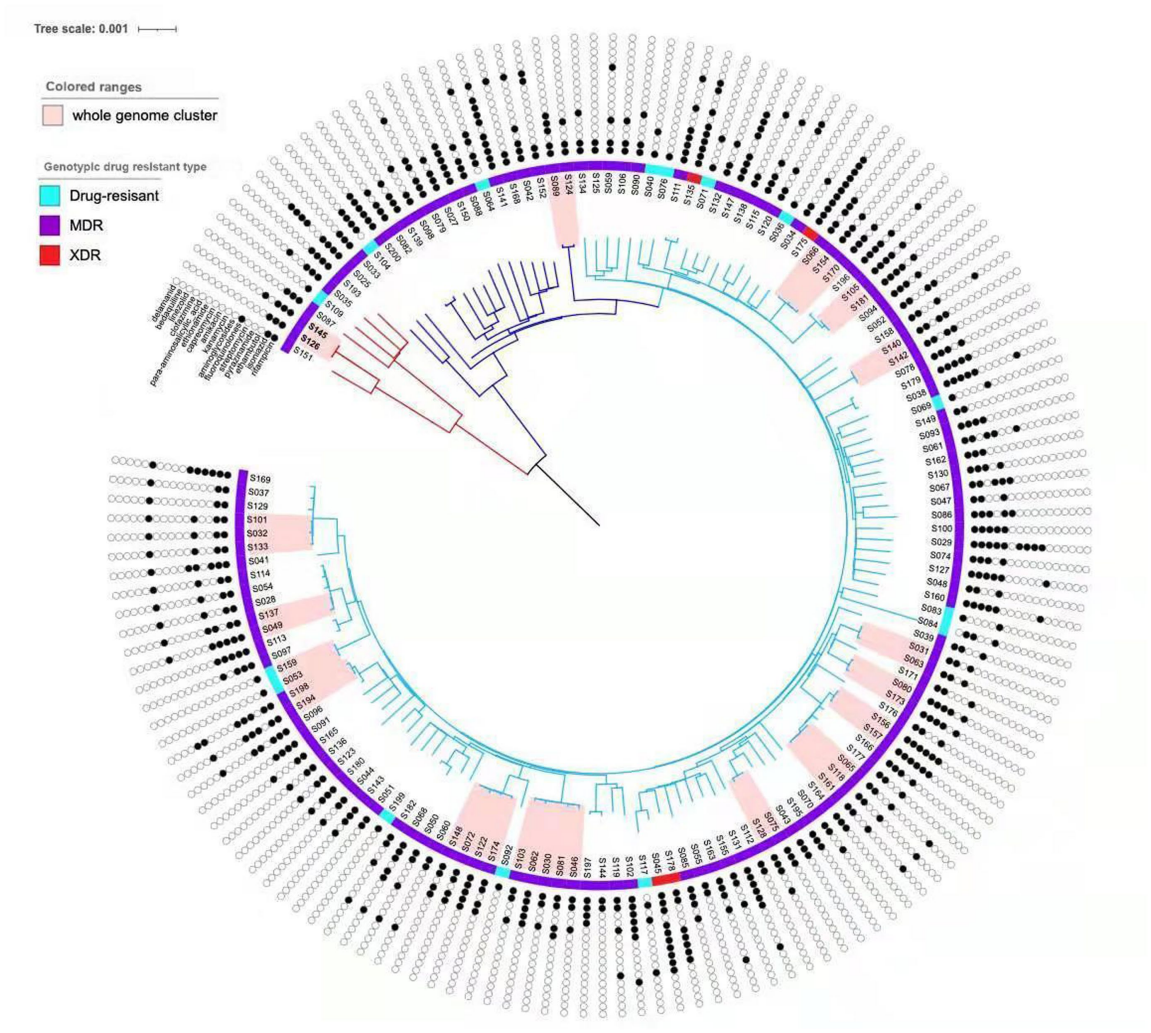
Phylogenetic tree of the 149 MTB isolates in this study. The sublineages, genotypic drug resistant type and drug resistant profile of the isolates are shown on the tree, according to the color legend shown on the left. The light pink bars indicate clustered isolates.

Analysis of the SNP distance matrix revealed 14 clusters comprising a total of 31 MDR-MTB isolates (Figure 2), resulting in a clustering rate of 20.81% (31/149) and a minimum estimate of recent transmission of 11.41%. Each cluster included 2–3 MDR-MTB isolates. Among these clustered isolates, 21 originated from new MDR-TB patients, yielding a clustering rate of 22.11% (21/95), while 10 isolates came from previously treated MDR-TB patients, with a clustering rate of 18.52% (10/54).

The clustering rate for lineage 4 (Euro-American) was 28.57% (2/7), compared to 20.42% (29/142) for lineage 2 (East Asian), though the difference was not statistically significant (χ^2^ = 0.0017, *p* = 0.9668) (Table 4). Within the clustered MDR-TB isolates, three pairs of patients resided within 2 kilometers of each other, including two pairs from the same household, suggesting direct transmission events.

**Table 4:** Comparison of clustering rates between lineage 2 and lineage 4.

Lineages	Clustered cases	Unclustered cases	χ2	*p*
Lineage 2 (East Asian)	29	113	0.0017	0.9668
Lineage 4(Euro-American)	2	5		

### Consistency of genotypic and phenotypic resistance in MDR-MTB strains

WGS determines drug susceptibility by identifying mutations in resistance-related genes. If such mutations are detected, the strain is classified as genotypically resistant; otherwise, it is considered genotypically susceptible. However, the agreement between WGS-predicted resistance and phenotypic drug susceptibility testing is not absolute.

For RIF, the compliance rate between WGS and phenotypic testing was 97.32% (145/149). The compliance rate for INH was 92.62% (138/149), while for OFX, it was 87.92% (131/149). SM had a compliance rate of 83.89% (125/149), and EMB exhibited the lowest compliance rate at 71.14% (106/149) (Supplementary Table S1).

## Discussion

In recent years, WGS has proven to be a highly accurate method for predicting resistance to most first- and second-line anti-TB drugs, offering high sensitivity and specificity (18). Compared with targeted molecular resistance detection methods like GeneXpert MTB/RIF, WGS provides several advantages, including comprehensive detection of all drug resistance-associated mutations, identification of potential novel mutations, and detection of heterogeneous drug resistance.

Globally, the majority of studies have shown that mutations within the 81-bp rifampin-resistance-determining region (RRDR) of the rpoB gene account for over 96.00% of RIF resistance (19). In this study, 97.93% of RIF-resistance mutations were located within the RRDR. Mutations outside the RRDR are often undetected by molecular methods such as GeneXpert MTB/RIF. Notably, this study identified three mutations (rpoB Glu761Asp., Phe424Val, and Thr400Ala) outside the RRDR, which have not been previously reported in the literature. Additionally, five compensatory mutations related to RIF resistance were identified in the rpoC gene.

For INH resistance, mutations in the fabG1 and katG genes were detected in 92.62% (138/149) of MDR-MTB isolates, underscoring their pivotal role in conferring resistance. The most frequent mutation in katG was Ser315Thr (62.42%, 93/149), while the most common mutation in fabG1 was c-15C > T (8.05%, 12/149). A previously unreported katG mutation (−7G > A) was identified, along with a rare inhA Ile194Thr mutation, which had been documented in a prior Indian study (6). Furthermore, a novel *ahpC* mutation (c-52C > T) observed in this study was consistent with findings from southern Brazil (20). Nine compensatory mutations in the ahpC gene were also identified, highlighting their potential role in mediating INH resistance. These mutations have been reported in several other studies (20, 21).

For EMB resistance, the most prevalent mutations identified in this study were *embB* Met306Val (42.86%), Met306Ile, and Asp354Ala. The proportion of the *embB* Met306Val mutation observed here was lower than that reported in a Chinese study (22) but higher than the findings from an Ethiopian study (23). Additionally, a single mutation in the *embA* gene was detected in one strain; however, this mutation was not identified by the GenoType MTB DRplus Version 1 assay (Hain Lifescience, Germany).

Regarding SM resistance, the most frequent mutation among MDR-TB patients was rpsL Lys43Arg (68.13%), consistent with studies conducted in Taiwan (24), Hainan (25), China (22, 26), and southern China (27). Mutations rrs 514a > c and 517c > t were also reported in previous studies by Zhao (28) and Dorji (29). Furthermore, a novel mutation, rpsL Lys43Thr, identified in Mejía-Ponce’s study, was associated with reduced fitness (30).

Fluoroquinolones are essential in the treatment of MDR-TB and serve as one of the core drugs in treatment regimens. Resistance to quinolones primarily arises from mutations in the gyrA and gyrB genes (31). In this study, the most frequent mutation observed was gyrA Asp94Gly (22.50%, 9/40), consistent with findings from several studies conducted in China (32, 33).

For injectable aminoglycosides, the rrs 1401A > G mutation is a critical determinant of resistance. This study revealed mutation rates of 100.00, 80.00, and 50.00% for AMK, KAN, and CAP, respectively.

ETO resistance is well-documented to be associated with the *fabG1* c-15C > T mutation (34). In this study, fabG1 c-15C > T and inhA mutations were the most prevalent, while one isolate exhibited an ethA mutation. The inhA Ile194Thr mutation, however, was not found in the WHO mutational catalog (35).

While PAS has been utilized in TB treatment for over 70 years, its mechanism of action remains incompletely understood (36). Resistance to PAS is generally linked to mutations in genes such as thyA, folC, and ribD (37). In this study, the most common mutation observed was *thyX* c-16C>T, corroborating findings from Srilohasin’s research (38). Additionally, folC Ser150Gly and Glu40Gly mutations were detected, similar to findings reported in Australia (29).

This study evaluated the efficacy of WGS in predicting drug resistance, using phenotypic DST as the reference standard. WGS demonstrated a high concordance rate (over 80.0%) for predicting resistance to RIF, INH, SM, and OFX, aligning with previous studies (39, 40). The discrepancy between WGS and phenotypic DST results may arise from several factors. First, phenotypic DST might fail to detect resistance in samples with low resistance levels (41). Second, WGS cannot capture resistance mechanisms beyond genetic mutations, such as those induced by efflux pumps (42). However, the accuracy of WGS in predicting EMB resistance was notably lower, consistent with other reports (25, 43). This lower accuracy may be due to single mutations, such as embA or embB M306I/G406A, being present in both EMB-resistant and EMB-sensitive strains (43, 44).

The genetic variations responsible for *Mycobacterium tuberculosis* drug resistance differ significantly across regions, influenced by factors such as national TB control policies, treatment strategies, population-related medical behaviors, and strain phylogenetic backgrounds (45–47).

In our study, the L2 and L4 lineages were the predominant strains among MDR-TB isolates from Wuhan, with L2 accounting for 95.30%. Of the L2 strains, 88.03% were classified as modern Beijing strains. The frequency of the embB Met306Ile mutation in L4 strains was higher than in L2 (*p* = 0.0150), while the frequency of the rpsL Lys43Arg mutation was lower in L4 strains compared to L2 (*p* = 0.0333). No significant differences were observed in mutation frequencies at other sites. The folC Ile43Thr mutation, associated with PAS resistance, was exclusively found in L2 strains, consistent with findings in other studies (26, 48, 49).

The Beijing genotype strains were further categorized into modern and ancient Beijing types (50, 51). Modern Beijing strains of MTB are more prevalent than ancient Beijing strains worldwide and have been responsible for the majority of TB outbreaks (52, 53). In our study, there were no significant differences in mutation types between modern and ancient Beijing strains, which contrasts with several previous studies (54), possibly due to the relatively small sample size. Additionally, the clustering rate between lineage 4 and lineage 2 strains did not show significant differences, consistent with studies conducted in Hainan (25) and Hunan (55), China.

WGS data of MDR-TB strains from Wuhan were used in this study to assess clustering based on SNP distance. A clustering rate of 20.81% was observed, with a minimum estimated proportion of recent transmission at 11.41%, which was lower than the findings reported by Yang (56) in Shanghai and similar to the results from Jiang (57) in Shenzhen.

The findings regarding Beijing family classification and drug-resistance-associated gene sequencing were consistent with our previous research (13). However, the clustering results differed: 38 cases were identified in 11 clusters using the DTM-PCR and MIRU-VNTR methods (13), whereas 31 cases were grouped into 14 clusters with WGS. Only one cluster (two cases) was identical across both genotyping methods. In this study, two groups of family members (each consisting of two individuals) were identified based on their residential addresses in the Wuhan TB reporting network, and both groups were clustered by WGS. In contrast, only one group was clustered by DTM-PCR and MIRU-VNTR (13).

Thus, the standard 24-locus MIRU-VNTR method has limitations due to the homology within strains of the Beijing family, and the combination of DTM-PCR and MIRU-VNTR did not adequately discriminate between strains to comprehensively trace transmission (12, 58, 59) provides more accurate gene clustering results in regions and countries where the Beijing family of *Mycobacterium tuberculosis* is predominant.

The recent transmission rate observed in this study may have been underestimated for several reasons. First, the MDR-TB patients enrolled were from Wuhan Pulmonary Hospital, excluding those from another designated hospital, Jin-yin-tan Hospital, which represents only approximately 40% of the total patient population in Wuhan. Second, the TB culture positivity rate in Wuhan in 2017 was only 40%, which may have limited the effectiveness of genotyping studies. Third, the study sample was collected in 2017, slightly earlier than anticipated due to delays caused by the Covid-19 pandemic. Consequently, newer anti-tuberculosis drugs, such as Bedaquiline and Delamanid, were not included in the treatment regimen. Furthermore, the epidemiological links among clustered patients were not investigated, partly due to loss of follow-up. As noted in previous studies (60, 61), the use of a single reference genome, H37Rv, may not adequately detect virulence-related loci in *Mycobacterium tuberculosis*. These five factors represent the main limitations of this study.

## Conclusion

This study is the first to employ WGS to detect drug resistance-related gene mutations and assess the genetic clustering of MDR-MTB strains, thereby providing valuable insights into the molecular epidemiology of MDR-TB in Wuhan, China. The genetic diversity of MDR-TB in Wuhan mirrors global trends but also exhibits distinct regional characteristics. MDR-TB demonstrates localized transmission within Wuhan. WGS has proven to be an effective tool for investigating the molecular epidemiology of MDR-TB in this region. The findings of this study may serve as a critical reference for the prevention and clinical management of MDR-TB in Wuhan.

## Data Availability

The datasets presented in this study can be found in online repositories. The names of the repository/repositories and accession number(s) can be found in the article/Supplementary material.

## Footnotes

^1^ https://github.com/jodyphelan/tbdb
